# Identification of Antigens Specific to Non-Tuberculous Mycobacteria: The Mce Family of Proteins as a Target of T Cell Immune Responses

**DOI:** 10.1371/journal.pone.0026434

**Published:** 2011-10-25

**Authors:** Anna M. Checkley, David H. Wyllie, Thomas J. Scriba, Tanya Golubchik, Adrian V. S. Hill, Willem A. Hanekom, Helen McShane

**Affiliations:** 1 The Jenner Institute, Nuffield Department of Medicine, Oxford University, ORCRB, Oxford, United Kingdom; 2 South African Tuberculosis Vaccine Initiative and School of Child and Adolescent Health, Institute of Infectious Diseases and Molecular Medicine, University of Cape Town, Cape Town, South Africa; 3 Department of Statistics, Oxford University, Oxford, United Kingdom; Institut de Pharmacologie et de Biologie Structurale, France

## Abstract

The lack of an effective TB vaccine hinders current efforts in combating the TB pandemic. One theory as to why BCG is less protective in tropical countries is that exposure to non-tuberculous mycobacteria (NTM) reduces BCG efficacy. There are currently several new TB vaccines in clinical trials, and NTM exposure may also be relevant in this context. NTM exposure cannot be accurately evaluated in the absence of specific antigens; those which are known to be present in NTM and absent from *M. tuberculosis* and BCG. We therefore used a bioinformatic pipeline to define proteins which are present in common NTM and absent from the *M. tuberculosis* complex, using protein BLAST, TBLASTN and a short sequence protein BLAST to ensure the specificity of this process. We then assessed immune responses to these proteins, in healthy South Africans and in patients from the United Kingdom and United States with documented exposure to NTM. Low level responses were detected to a cluster of proteins from the mammalian cell entry family, and to a cluster of hypothetical proteins, using *ex vivo* ELISpot and a 6 day proliferation assay. These early findings may provide a basis for characterising exposure to NTM at a population level, which has applications in the field of TB vaccine design as well as in the development of diagnostic tests.

## Introduction

Tuberculosis (TB) remains a major threat to global public health, with an estimated 9.27 million new cases occurring worldwide in 2007 [1]. TB incidence rates are particularly high in countries with a high HIV prevalence, and South Africa alone accounts for 25% of the worldwide HIV-associated TB burden [2]. Increasing rates of drug resistance add to the difficulty of treatment, particularly in resource-poor settings. An effective TB vaccine would represent the most cost-effective approach to global TB control [3]; there are currently several new candidate TB vaccines under development.

BCG is currently the only licensed TB vaccine. It protects against severe forms of the disease in childhood, but has very poor efficacy in preventing adult pulmonary TB where it is most needed, in tropical countries which have a high incidence of TB. Protection ranges from 80% in the UK [4] to 0% in Malawi [5]; 41% of this variability has been attributed to the latitude at which the study was conducted [6].

One theory as to why BCG works less well in the tropics than in temperate regions is exposure to non-tuberculous mycobacteria (NTM) [7]. Animal models have shown that mice exposed to *M. avium* had a reduced protective immune response to subsequent BCG vaccination, cleared the live BCG vaccine more rapidly than mice not exposed to *M. avium*, and were more susceptible to *M. tuberculosis* infection following BCG vaccination [8]. A model in which *M. avium* was administered after BCG vaccination showed declining efficacy of BCG with ongoing exposure to *M. avium* [9]. Conversely, other animal models have shown partial protection against *M. tuberculosis* infection following NTM exposure [10], [11], and variations in dose [12], species [8] and strain [11], [13] of infecting NTM or of *M. tuberculosis* [14] have been shown to influence whether NTM exposure interferes with BCG efficacy or contributes towards protection against M. tuberculosis. It is not known whether, if exposure to NTM has an effect on the efficacy of BCG, this is caused by those antigens common to both BCG and NTM (which may provide a cross-protective effect), or by those antigens which are present in NTM but not in BCG (which could produce an antagonistic effect).

In humans, Black *et al* have shown that adolescents living in Malawi had marked pre-existing T cell responses to purified protein derivative (PPD) prior to BCG vaccination. After vaccination with BCG the increase in response to PPD was minimal, in contrast to adolescents in the UK, in whom baseline responses were very low and a marked increase in PPD-specific responses post BCG-vaccination was detected [15]. These pre-vaccination immune responses to PPD were attributed to NTM exposure; the most likely explanation, given that individuals were BCG naïve, and those with a tuberculin skin test result suggestive of TB exposure had been excluded from the study.

It is, however, hard to characterise NTM exposure with certainty, as there are currently no defined antigens which are specific to NTM and not also present in (and therefore confounded by exposure to) *M. tuberculosis* and BCG. Although previous studies have investigated NTM exposure using PPD derived from NTM species [16], approximately 80% of proteins are shared between PPDs of different species of NTM and M tuberculosis, so it is hard to definitively attribute an immune response to any particular species.

Further, the nature of the T cell response necessary for protection against mycobacterial infection is not known: a limited number of strong responses to critical epitopes or antigens may be required; alternatively multiple low affinity T cell responses to various antigens may provide protection, including cross-species protection.

If exposure to NTM has an effect on BCG replication and hence immunogenicity and protective efficacy, this effect might also be seen with novel TB vaccines based on BCG. Viral vectored and protein/ adjuvant subunit vaccines do not replicate, so may not be susceptible to interference by this mechanism [8]. It will be important in the development of new TB vaccines to ensure that their efficacy is not reduced by exposure to NTM, and in order to do this, specific measures of NTM exposure are needed. NTM-specific antigens could also be of diagnostic use to the veterinary and medical fields. NTM infections (for example, *M. paratuberculosis*) cause considerable morbidity and economic losses in animal husbandry [17] and also cause human disease in certain situations.

In this study, we aimed to define antigens which are specific to NTM (i.e. not present in the *M. tuberculosis* complex), and which could be used to study NTM exposure with a high degree of specificity. This is relevant for studies both of BCG efficacy and of novel TB vaccines currently in development. We used a bioinformatic approach to define families of proteins which are present in common NTM and not in the *M. tuberculosis* complex, and investigated the T cell immune response to these antigens in patients in the UK and US who had been exposed to NTM, in cord blood samples with a low chance of NTM exposure, and in healthy South Africans from the Western Cape, an area with documented NTM exposure [18].

## Methods

### Bioinformatic methods

All available mycobacterial genome and reference protein sequences were downloaded from the National Center for Biotechnology Information (NCBI) and Sanger websites on 2 December 2009 and stored in a BioSql database [19], [20], [21]. Sequence information from our Illumina GAIIx next generation sequencing of a *M. fortuitum* isolate (isolate submitted to ATCC, ID awaited, sequence uploaded to NCBI, ID awaited), was also loaded into the database. Mycobacterial species were classified into 3 groups: group 1 =  NTM species of interest, group 2 = *M. tuberculosis* complex, group 3 =  all other mycobacterial species (Table 1). NTM species of interest were defined as those reported to be frequently isolated in environmental and clinical studies from the UK and South Africa [18], [22], [23], [24], [25], [26], [27]. The Prodigal tool (v1.1), which performs well in high GC content genomes, was used to predict open reading frames and protein sequences on all incomplete and un-annotated genomes [28], [29].

**Table 1:** Genome sequences downloaded.

Group 1: NTM of interest	Group 2: *M. tuberculosis* complex	Group 3: Other mycobacteria
*M. avium 104*	*M. tuberculosis^6^*	*M. gilvum*
*M avium paratuberculosis K10*	*M. africanum^1,7^*	*M. leprae* ^10^
*M. intracellulare^1,2^*	*M. bovis^8^*	*M. marinum*
*M. kansasii^1,3^*	*M. bovis* BCG^9^	*M. smegmatis*
*M. fortuitum^4^*		*M. ulcerans*
*M. absessus^5^*		*M. vanbaalenii*
		*Mycobacterium JLS*
		*Mycobacterium KMS*
		*Mycobacterium MCS*

These genome sequences were downloaded from the NCBI website [37], unless otherwise stated. Sequences are divided into NTM of interest (group one), *M. tuberculosis* complex (group two) and other mycobacterial species not of interest to the project.

1. Incomplete sequences,

2. *M. intracellulare* ATC 13950,

3. *M. kansasii* ATCC 12478,

4. next generation sequencing, Oxford University,

5. Genoscope,

6. *M. tuberculosis* CDC1551, *M. tuberculosis* F11, *M. tuberculosis* H37Ra, *M. tuberculosis* H37Rv sequences,

7. Sanger website[38],

8. *M. bovis* AF2122/97,

9. *M. bovis* BCG str. Pasteur 1173P2, *M. bovis* BCG [Fiocruz - FAP],

10. *M. leprae* Br4923, *M. leprae* TN.

NCBI protein BLAST was used to compare all protein sequences in the database against each other using NCBI default parameters [30]. This approach was validated by demonstrating identification of the *M. tuberculosis*-specific RD1 gene products [31] from a BLASTP comparison of predicted proteins from *M. tuberculosis* CDC1551 and *M. bovis* BCG (data not shown).

A protein was selected if present in at least 3 other species from group 1 (NTM of interest) and absent from group 2 (*M. tuberculosis* complex). Selected proteins were subsequently compared with all group 2 mycobacterial genome sequences using TBLASTN, and excluded if significant matches were found. NCBI BLASTClust [32] was used to arrange the selected proteins into clusters. Following optimisation on known families, ≥50% identity over ≥50% length cut-offs were selected. The predicted cellular location of clustered reference proteins were noted based on the locateP prediction algorithm [33].

Clusters were selected for experimental testing if they: (1) contained no proteins which hit nucleotide sequences in group 2 (*M. tuberculosis* complex) during TBLASTN, or (2) either (a) contained a majority of proteins with a prediction to be secreted or (b) contained proteins for which there was experimental evidence of immunogenicity.

Clusters were aligned using ClustalW [34] with default parameters, and ends were manually trimmed. This was done because it was found that sometimes the Prodigal tool selected initiator sites which were substantially upstream of the canonical start site of other family members.

20-mer peptide sequences overlapping by 10 amino acids were generated computationally. A further BLASTP was carried out with these sequences against all bacterial reference protein sequences. An exact hit of 9 consecutive amino acids was considered significant. Peptides with significant hits against members of the *M. tuberculosis* complex or common species of other bacteria were excluded.

### Study populations

Ethical approval was obtained from the University of Cape Town, South Africa (REC reference 126/2006), from Oxfordshire, UK (REC B, reference 09/H0605/75) and from Portland VA Medical Center, Portland, United States (US) (reference IRB00004835). Written informed consent was obtained from all study participants. Healthy individuals living in the Western Cape, South Africa, who were known to have a negative result to the QuantiFERON®-TB Gold In-Tube test (Cellestis) were recruited. An additional cohort of individuals who were known to have a positive result was also recruited. Patients from whom NTM had been isolated from sputum on at least 2 occasions (or at least once from bronchoalveolar lavage) and with a low risk of TB exposure were recruited from the Churchill Hospital, Oxford, UK, and from Portland VA Medical Center, US. Cord blood was collected at the John Radcliffe Hospital, Oxford, UK.

### Immunology

#### Peptides

Peptides were dissolved in DMSO, stored at 1 mg/ml at −20°C and used at a final DMSO concentration of <0.35%. Pools were arranged such that each pool contained peptides from only one cluster except in the case of very small clusters, which were combined (Table 2).

**Table 2:** Protein clusters selected for the generation of peptides.

Cluster id	Protein family	Number of proteins/ cluster	Ratio secreted prediction/ intracellular	Peptide pool number
**A**	Mce family protein	16	5	1–5
**B**	hypothetical protein	13	5	6–7
**C**	hypothetical protein	12	5	8,11
**D**	hypothetical protein	8	5	9
**E**	hypothetical protein	8	5	10
**F**	hypothetical protein	7	4	11,12
**G**	hypothetical protein	7	4	13
**H**	hypothetical proteins	7	4	17
**I**	Tat-translocated enzyme	6	4	14,15
**J**	27 kDa lipoprotein antigen	6	4	16
**K**	hypothetical protein	6	4	17
**L**	hypothetical protein	5	3	17

Protein clusters were tested in the form of overlapping peptides, shown by cluster id, protein family to which they belong, number of proteins per cluster, the ratio of proteins with a prediction to be secreted over those predicted to be intracellular and the number of the peptide pool in which those proteins were tested. Peptide pools 18–20 consisted of peptides which hit bacterial reference proteins with a low affinity on protein BLAST. These peptides came from all clusters, and were tested separately from the others as there was a concern they may be less specific.

#### *Ex vivo* IFN-γ ELISpot assay

50 ml blood was taken from adult volunteers into sodium heparin tubes. Cord blood was taken into a standard blood donor collection bag, containing citrate phosphate dextrose anticoagulant. Peripheral blood mononuclear cells (PBMC) were separated and cryopreserved from the UK blood samples as previously described [35]; those from South African volunteers were used immediately. PBMC were thawed prior to use and rested overnight in 10 U/ ml benzonase nuclease (Novagen) at 37°C 5% CO_2_ in a humidified incubator [35].

PBMC IFN-γ responses to NTM-specific peptides (ProImmune, UK, 20-mers overlapping by 10 amino acids, 4 µg/ml), PPD (positive control, Statens Serum Institut, 10 µg/ml), phytohaemagluttinin/ phorbol 12-myristate 13-acetate (PHA/PMA, positive control, 10 µg/ml/ 50 µg/ml) and pool of ESAT-6/ CFP-10 peptides ((negative control), 15-mers overlapping by 10 amino acids, 4 µg/ml) were measured using overnight *ex vivo* ELISpot assay. Peptides were tested in 20 pools, each pool containing between 58 to 85 peptides. Briefly, nitrocellulose bottomed 96-well Multiscreen HA filtration plates (Millipore, UK Ltd) were coated with anti-human-IFN-γ-mAb (Mabtech, UK) overnight at 4°C. 3×10^5^ PBMC were plated in 100 µl final volume and plates were incubated for 18–20 h in a humidified 37°C 5% CO_2_ incubator with peptide, PPD, PHA/PMA or media alone. Assays were performed in duplicate and the results averaged. Plates were washed and developed as previously described [35]. A plate was considered to have passed if there were at least 200 spot forming cells (SFC)/ 10^6^ PBMC in at least one positive control well, and if both negative control wells had <20 SFC. Cut off for a positive response was calculated as 3 median absolute deviations (MADs) above the median.

#### Proliferation assay

All cryopreserved PBMC samples from UK-based NTM-exposed patients and cord blood samples were analysed, where remaining cell numbers allowed [36]. Cells were incubated with media alone or PPD (2 µg/ml), NTM-specific peptide pool number 5, 6, 11 or 17 or ESAT-6/ CFP-10 pool (all 1 µg/ml) for 6 days at 37°C with 5% CO_2_. On day 3, PHA (1 µg/ml) was added to one of the ‘media only’ wells. On day 6, PBMC were stained with LIVE/DEAD Fixable Violet Dead Cell Stain (Invitrogen) and fixed with BD FACS Lysing Solution (BD Biosciences). Following permeabilisation with Perm/Wash (BD Biosciences) cells were incubated with the following monoclonal antibodies: anti-CD3-QDot 605, anti-CD4-APC, anti-CD8-PerCP-Cy5.5 and anti-Ki67-PE. All antibodies were from BD Biosciences except for CD3-QDot 605, which was from Invitrogen, and were used in pre-determined optimal concentrations. Following washes, samples were acquired on a BD LSRII flow cytometer (BD Biosciences, San Jose, CA). Time gates (excluding fluctuations in fluorescence), antibody aggregate exclusion gates and forward scatter/ side scatter gates (selecting singlets) were followed by gating on live CD3 positive cells, then CD4 or CD8 positive cells, then Ki67 positive cells. Data were analysed using FlowJo Software version 8.8.3 (Treestar Inc.) and GraphPad Prism (version 5). Proportion of Ki67 positive was used as the readout of proliferation [36]. A sample was considered to have passed if percentage proliferating cells from the un-stimulated control was less than 2%, if proliferation to either PPD or to PHA was greater than 10% and if proliferative responses to ESAT-6/CFP-10 peptides was less than 2%. In addition to frequency of proliferating cells, the stimulation index was calculated as response in test well/ response in un-stimulated well; a SI greater than 2% was considered positive.

## Results

### Selection of NTM-specific proteins

**Figure 1:**
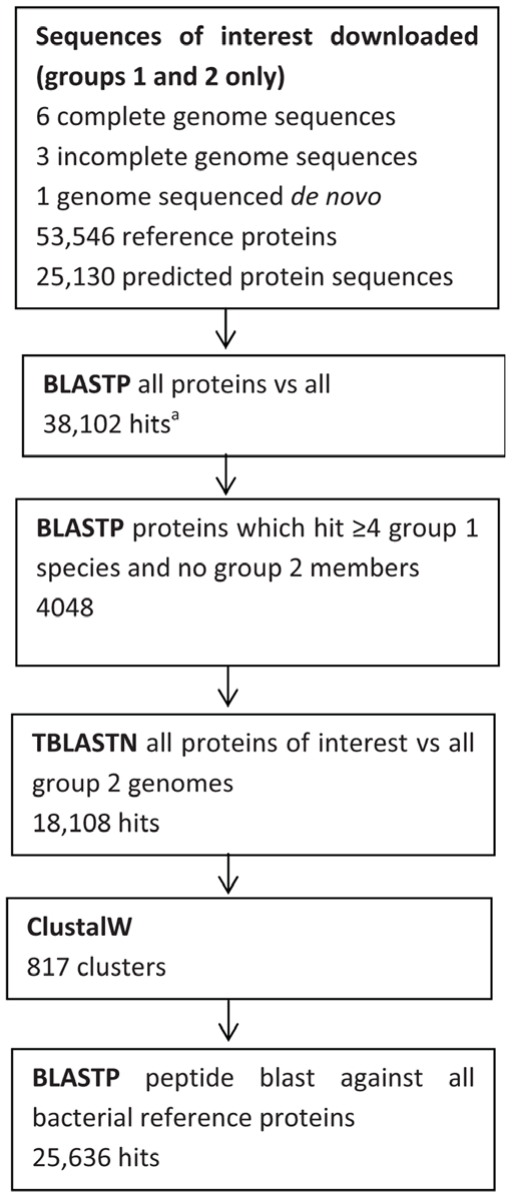
Bioinformatic pipeline. Summary of the steps involved in the bioinformatic pipeline, showing numbers of sequences, hits and clusters generated. a: hits between proteins from group 1 or 2 only (group 3 hits excluded). BLASTP: protein BLAST, TBLASTN: BLAST of protein sequence against 6-frame translated nucleotide sequence, ClustalW: protein clustering tool [34].

Fifteen complete and annotated mycobacterial genome sequences [37], three incomplete mycobacterial sequencing projects [38] and 102,051 associated reference proteins [21] were available for analysis (Table 1, Figure 1). Species were classified into 3 groups: NTM of interest (group one; isolates commonly recovered from UK and South African clinical and environmental samples [18], [22], [23], [24], [25], [26], [27]), *M. tuberculosis* complex (group two) and all other mycobacterial species (group three) (Table 1).

### Open reading frame prediction and *M. fortuitum* sequencing

Next-generation (Illumina GAIIx) sequencing was performed on a clinical *M. fortuitum* isolate, and genome assembly was undertaken using the programme Velvet [39]. The open reading frame prediction tool Prodigal (v1.1) [29] was applied to all contigs from incomplete sequencing projects (including *M. fortuitum*), and predicted protein sequences were derived (Figure 1). Median predicted protein length was 243, 235, 275 and 185 amino acids for the *M. intracellulare*, *M. kansasii*, *M. africanum* and *M. fortuitum* genomes (median contig lengths 7888, 9278, 98,860 and 2528 respectively).

NCBI protein BLAST was used to compare all protein sequences against themselves (see methods); 4048 group 1 proteins hit proteins from at least 3 other mycobacterial species in group 1 genomes (NTM species of interest), and none in group 2 (*M. tuberculosis* complex). These proteins were selected for further analysis.

### Clustering of mycobacterial proteins and selection of NTM-specific proteins

BLASTClust was used to cluster these proteins into related families. Any cluster which contained a protein that had hit a member of the *M. tuberculosis* complex during the TBLASTN process was excluded, leaving 78 clusters varying in size from 2–17 proteins. These clusters were biologically relevant, containing families such as the Mce family, the DoxX and the 27kDa lipoprotein antigen. From these, 12 clusters were selected for experimental testing (Table 2). 11 were prioritised on the basis that they were predicted to contain predominantly secreted proteins, and one (Mce family proteins) on the basis of reports of immunogenicity [40], [41].

### Stringent exclusion of peptides also present in *M. tuberculosis* complex and common bacteria

Protein BLAST (see Methods) was performed on all computationally defined peptide sequences against all published bacterial reference protein sequences, resulting in 25,636 hits (Figure S1). Peptides with hits to non-mycobacterial reference proteins were eliminated from the process and 1699 peptides were synthesised.

### T cell responses to Mce family proteins

**Figure 2:**
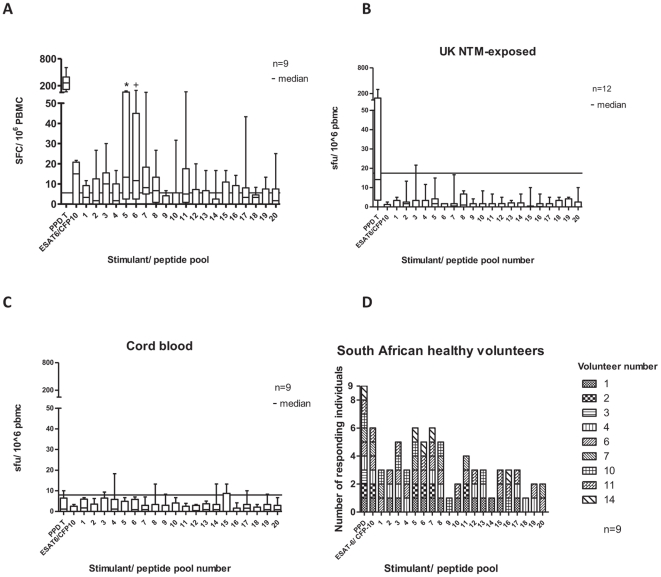
IFN-γ *ex vivo* ELISpot responses to pools of NTM-specific peptides. IFN-γ *ex vivo* ELIspot assay comparing T cell responses in PBMC from healthy South Africans (A), NTM-exposed UK patients (B), UK cord blood samples (C) and number of responding South African healthy volunteers to each peptide pool (D). * P = 0.06, + P = 0.02, Wilcoxon matched pairs test, comparing peptide response with unstimulated cells. No other responses were statistically significantly different from unstimulated. Assay cut off  = 3 median absolute deviations (MADs) above the median response to unstimulated PBMC. Median response (line), interquartile range (box) and range (whiskers) shown. Cut offs were 5 (A), 17.5 (B) and 8.33 SFC/ 10^6^ PBMC (C, Wilcoxon matched pairs test).

PBMC IFN-γ responses to PPD, pool of ESAT-6/ CFP-10 peptides and pools of investigational peptides were determined by *ex vivo* IFN-γ ELISpot assay in 9 healthy South African donors with previous negative responses to QuantiFERON®-TB Gold In-Tube (Figure 2). Responses to PPD were universally strong, and some of these individuals had low level responses to the ESAT-6/ CFP-10 peptide pool. Analysing responses to NTM-specific proteins, responses to peptide pools 5 and 6 were significantly increased above the negative control (Wilcoxon signed rank test for matched pairs). In addition, a broad range of individuals made responses over the cut off, in particular to pools 3, 5 (from cluster OA, the MCA family of proteins), 6, 7 (from cluster OB, a family of hypothetical proteins of unknown function) and pool 8 (from cluster OC, another family of hypothetical proteins of unknown function). Analysing results from the South African cohorts with negative and positive responses to QuantiFERON®-TB Gold In-Tube together (16 individuals), there was no significant correlation between response to any NTM-specific peptide pool and to the ESAT-6/ CFP-10 pool of peptides (Figure S2).

IFN-γ ELISpot was performed on 14 UK patients known to have had NTM isolated from clinical specimens, using previously frozen PBMC. In contrast with the healthy South African subjects, these individuals had a high prevalence of co-morbidities (Table 3). Two individuals had low level responses to the ESAT-6/ CFP-10 peptide pool and were excluded from further analysis, on the basis of possible TB exposure in the past. Among the remaining 12, the magnitude of response to PPD was low, and there were no statistically significant responses to any of the experimental peptide pools (Figure 2B). There were no statistically significant responses to either PPD or peptide pools in the 9 ‘negative control’ cord blood samples collected in the UK.

**Table 3:** Clinical characteristics of patients exposed to NTM.

No.	Age/years	Sex	Country	Relevant diagnoses	Organism isolated	Treated?	Steroids?
**1**	21	M	UK	Cystic fibrosis	*M kansasii, M abscessus*	Y^a^	Y
**2**	41	F	UK	Bronchiectasis	*M fortuitum*	N	Y
**3**	22	F	UK	Cystic fibrosis	*M chelonae, M abscessus*	Y^a^	Y^b^
**4**	88	M	UK	Bronchiectasis	*M gordonae*	N	N
**5**	28	M	UK	Cystic fibrosis	*M chelonae*	Y	N
**6**	59	F	UK	Bronchiectasis	*M chelonae, M xenopi*	N	N
**7**	28	M	UK	Cystic fibrosis	*M avium*	N	N
**8**	37	F	UK	Cystic fibrosis	*M abscessus, M chelonae*	N	N
**9**	72	M	UK	Bronchiectasis	*M chelonae, M gordonae, M fortuitum*	N	Y^c^
**10**	72	F	UK	Bronchiectasis	*M intracellulare*	N	N
**11**	57	F	UK	Bronchiectasis	*M avium*	Y	N
**12**	75	M	UK	COPD^d^	*M malmoense*	N	Y
**13**	27	M	UK	Bronchiectasis	*M intracellulare*	N	N
**14**	76	M	UK	Bronchiectasis	*M avium*	Y	N
**MB10**	76	F	US	Bronchiectasis	*M avium intracellulare*	Y	N
**MB11**	65	F	US	Bronchiectasis	*M avium intracellulare*	Y	Y
**MB16**	64	F	US	Bronchiectasis	*M avium intracellulare*	Y	N
**MB40**	78	F	US	Corticosteroid use	*M avium intracellulare*	N	Y
**MB41**	81	F	US	Bronchiectasis	*M avium intracellulare*	N	N

Summary of the clinical characteristics of patients exposed to NTM. M = male, F = female, Y = yes, N = no, Steroids = individual taking steroids currently or within past 6 months. a. low level of adherence to prescribed treatment, b. very recent (2 doses only), c. very low dose (1 mg prednisolone per day), d. chronic obstructive pulmonary disease.

**Figure 3:**
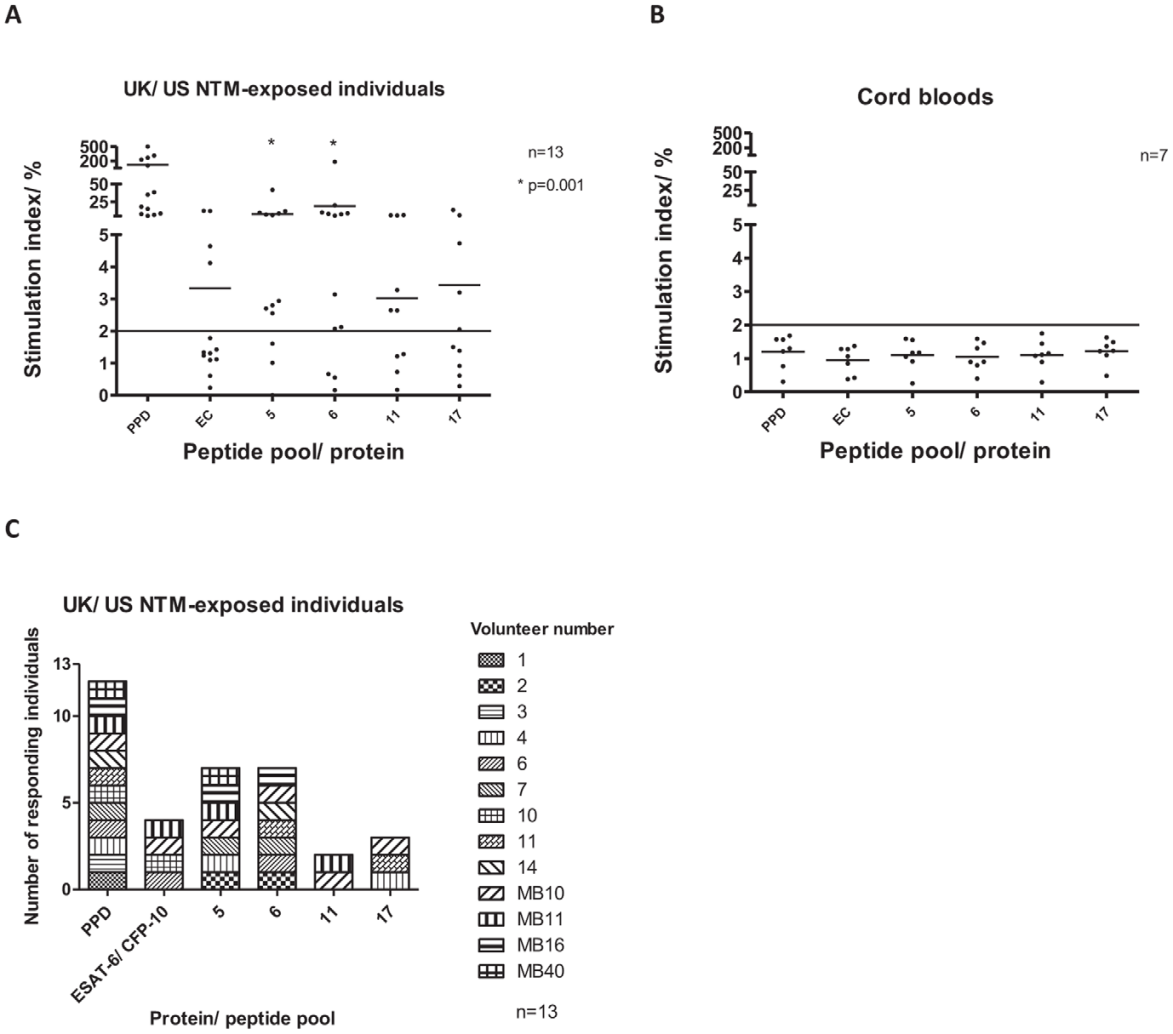
6 day proliferation assay responses to pools of NTM-specific peptides. Percentage Ki67 positive proliferating CD4 + lymphocytes in UK and US NTM-exposed patients (A), UK cord blood samples (B) and number of responding UK and US patients to each peptide pool (C). Assay cut off  =  stimulation index of 2%. Stimulation index  =  response in test well/ response in un-stimulated well.

A 6 day proliferation assay [42], which may be more sensitive than *ex-vivo* ELISpot, was performed on 18 samples from the UK and the US; 5 were excluded for technical reasons, leaving 13 for analysis (see Methods, Table 3). The proliferation assay used four NTM-specific peptide pools: 5, 6, 11 and 17, chosen on the basis of significant responses (pools 5 and 6) or borderline responses (pools 11 and 17) detected by ELISpot (Figure 2A). There were significant, high level proliferative responses to pools 5 and 6 in the UK based NTM exposed patients, with 10 individuals responding to both pool 5 and to pool 6 (Figure 3). CD8 T cell responses were not significant, and cord blood responses were again low. The amino acid sequences for peptides from pools 5 and 6 are shown in Figure S3, together with the identities of the proteins making up the clusters in these pools. Clusters A and B, from which pools 5 and 6 were derived, are shown in Figure S4.

## Discussion

We used a bioinformatic pipeline to define peptides which are shared between common NTM, eliminating peptides with sequence homology to either the *M. tuberculosis* complex or to common bacterial species. Protein BLAST was used to define an initial shortlist of proteins which are present in at least 4 common NTM (group 1 organisms) and not in the *M. tuberculosis* complex (group 2 organisms). TBLASTN, which is independent of genome annotation, was then used to increase the specificity of the results, comparing protein sequences against translated nucleotide sequences. This was done as protein sequences derived from genome annotation are not always experimentally confirmed, so accuracy of annotation may vary [43]. Finally, protein BLAST excluded the possibility of short sequence matches between NTM-specific peptides and the *M. tuberculosis* complex or common bacterial peptides. We focussed on secreted proteins since this group of proteins is associated with the induction of strong immune responses in mycobacteria [44], [45], [46], [47].

Using *ex vivo* IFN-γ ELISpot, statistically significant responses were seen to pools 5 and 6 of the 20 NTM-specific peptide pools in the healthy South African cohort. These pools represent the Mce family of proteins (pool 5) and a cluster of hypothetical proteins (pool 6, Figure 2). Additionally, a range of low-level responses was seen to multiple other peptide pools. Although similar responses were not detected using IFN-γ ELISpot in UK NTM-exposed patients, use of a possibly more sensitive proliferation assay demonstrated responses to peptide pools 5 and 6 in UK and US NTM-exposed patients. It may also be that the proliferation assay detected a different subset of T cells such as memory cells, rather than the effector cells that the overnight *ex vivo* ELISpot assay would be expected to detect.

The immunogenic Mce (mammalian cell entry) family of proteins are virulence factors which are involved in mycobacterial entry and survival in macrophages [48], [49]_ENREF_45. Mce knockout mutants of *M. tuberculosis* have reduced virulence in mice [50], *M. bovis* Mce4 induces an inflammatory response in bovine macrophages [40] and antibodies have been demonstrated to Mce proteins in TB-infected humans [41]. Importantly, the cluster of NTM-specific Mce proteins we selected is not similar in primary sequence to those in the *M. tuberculosis* complex; if it had been, these proteins would have been excluded from the pipeline. The remaining protein families against which immune responses were detected were hypothetical proteins of unknown function, which are the most frequent class of protein predicted by this, and by other similar pipelines [51], [52]. It is interesting that low-level responses were detected to such a wide range of peptide pools in the South African cohort. Such a pattern of responses, if present across the many antigens shared between NTM and *M. tuberculosis,* might contribute to a level of cross-protective immunity against *M. tuberculosis.*

It is promising that we appear to have demonstrated responses to NTM-specific antigens. However, responses were significantly lower than are routinely seen to immunodominant *M. tuberculosis-*associated mycobacterial antigens in individuals with latent *M. tuberculosis* infection. There are a number of possible explanations for this. Unlike *M. tuberculosis*, NTM are not highly pathogenic. About 80% of the predicted proteome of group 1 NTM is shared by members of the *M. tuberculosis* complex, and it is possible that these ‘core proteins’ of all mycobacteria are the most immunogenic. Secondly, in the absence of a gold standard test for NTM exposure we could not determine whether all South African volunteers had been exposed, nor how recently. The individuals in whom we detected positive responses may have been the only individuals with recent and significant exposure. Proliferative studies in a larger cohort with prospective assessment of NTM exposure might address this, although this would be associated with significant challenges. In the absence of a defined clinical phenotype, it remains extremely difficult to define populations of individuals with NTM exposure, and this is a significant limitation in the conduct of studies such as this.

Responses seen in UK and the US patients, where the exposure was definite but the duration of the NTM exposure cannot be quantified, were weaker than in South Africans. There are several possible explanations for this. These individuals suffered from chronic lung diseases, some of which are associated with low nutritional state and immune dysregulation [53] (Table 3). Supporting this, responses to PPD, a mixture of highly immunogenic proteins, were low compared to levels that would be expected in TB-exposed or recently BCG-vaccinated individuals [35], [54]. Additionally, assays in this group were performed on cryopreserved cells, in which responses may be reduced [55].

Bioinformatic pipelines have been used for the purposes of identifying antigens for the diagnosis of *M. bovis* infection in cattle [51] and leprosy [56] and *M. ulcerans* [57] in humans. The *M. bovis* pipeline consisted of a genome BLAST using the NCBI and Tuberculist servers. Cross-reactivity occurred between *M. bovis*-infected and BCG-exposed cattle, and further examination of the individual peptides responsible for cross-reactive responses highlighted that cross-reacting peptides hit similar sequences from *M. tuberculosis* on protein BLAST. Similarly, in the *M. ulcerans* pipeline, 11 out of 34 protein clusters identified using BLASTClust were found on PCR to have previously unknown homologues in strains of *M. marinum.* The *M. leprae* pipeline compared the *M. leprae* genome and predicted protein sequences with genome and predicted protein sequences of other published mycobacteria using BLAST and FASTA. Proteins were recognised by T cells from patients infected with M. tuberculosis as well as by those with leprosy [58], again suggesting cross-reactivity. Our bioinformatic elimination of potentially cross-reactive proteins, which was greatly aided by the increasing availability of NTM sequence data, appears to have achieved high levels of specificity. Of note, ESAT-6 and CFP-10 are present in *M. kansasii, M. szulgai* and *M. marinum*, but these species were not isolated from samples from any of the UK or US patients; we do not know which species the South Africans were exposed to.

In conclusion, we used a novel, comprehensive and stringent approach to define clusters of proteins which are predicted to be secreted and are present in common species of NTM but absent from *M. tuberculosis*, BCG and other members of the *M. tuberculosis* complex. In South Africans, we detected low level T cell responses to multiple proteins, including the Mce family of proteins, which are virulence factors in mycobacteria. Mce proteins and a pool from a cluster of hypothetical proteins were also recognised using a proliferation assay on PBMC from UK and US patients with NTM isolated from sputum samples. Further exploration of this approach is warranted, and the specificity of these promising pools could be confirmed by investigating larger cohorts of individuals from rural tropical areas with NTM exposure defined by surrogates such as strong PPD responses in the absence of any response to RD1 antigens such as ESAT-6 / CFP-10.

## Supporting Information

Figure S1 **Peptide ‘hits’ on bacterial reference proteins following Protein BLAST.** Protein BLAST was carried out on all peptide sequences against all bacterial reference proteins. The resulting hits are shown by bacterial genus. Y axis: median number peptide hits per genus. X axis: bacterial genus.
(TIF)

Figure S2 **Correlation between response to ESAT-6/CFP-10 peptide pool and NTM-specific peptide pools.** Correlation between response to pool of ESAT-6/ CFP-10 peptides and NTM-specific peptide pools, in all healthy South African volunteers.
(TIF)

Figure S3 **Peptide pools 5 and 6: constituent proteins and peptides.** Showing the identities of proteins making up the clusters in pools 5 and 6, and the amino acid sequences for the peptides derived from them.
(PDF)

Figure S4 **Protein clusters to which immune responses were detected using** ***ex vivo*** **ELISpot and proliferation assays.** A. Protein sequences making up cluster A: NP_960785.1: hypothetical protein MAP1851 [Mycobacterium avium subsp. paratuberculosis K-10], YP_881588.1: mce related protein [Mycobacterium avium 104], NZ_ABIN01000058_P_11090: predicted protein sequence from *M. intracellulare* genome, YP_001852130.1: Mce protein, Mce5A [Mycobacterium marinum M], YP_907368.1: Mce protein, Mce5A [Mycobacterium ulcerans Agy99], NOTNCBI_FOR1052_P_8286: predicted protein sequence from *M. fortuitum* genome, YP_879398.1: mce related protein [Mycobacterium avium 104], NP_959042.1: hypothetical protein MAP0108 [Mycobacterium avium subsp. paratuberculosis K-10], NZ_ABIN01000014_P_12211: predicted protein sequence from *M. intracellulare* genome, YP_001705239.1: putative Mce family protein [Mycobacterium abscessus ATCC 19977], YP_001701754.1: putative MCE family protein [Mycobacterium abscessus ATCC 19977], YP_001705291.1: putative Mce family protein [Mycobacterium abscessus ATCC 19977], YP_001848502.1: MCE-family protein Mce6A [Mycobacterium marinum M], YP_908277.1: MCE-family protein Mce6A [Mycobacterium ulcerans Agy99], YP_001702434.1: putative Mce family protein [Mycobacterium abscessus ATCC 19977], YP_001705322.1: putative Mce family protein [Mycobacterium abscessus ATCC 19977]. B. Protein sequences making up cluster B: YP_001852137.1: hypothetical protein MMAR_3871 [Mycobacterium marinum M], YP_907374.1: hypothetical protein MUL_3803 [Mycobacterium ulcerans Agy99], NP_960792.1: hypothetical protein MAP1858 [Mycobacterium avium subsp. paratuberculosis K-10], YP_881582.1: hypothetical protein MAV_2381 [Mycobacterium avium 104], NZ_ABIN01000058_P_11097: predicted protein sequence from *M. intracellulare* genome, YP_001848485.1: hypothetical protein MMAR_0160 [Mycobacterium marinum M], YP_908294.1: hypothetical protein MUL_4936 [Mycobacterium ulcerans Agy99], YP_001701747.1: hypothetical protein MAB_1003c [Mycobacterium abscessus ATCC 19977], NP_959049.1: hypothetical protein MAP0115 [Mycobacterium avium subsp. paratuberculosis K-10], YP_879405.1: hypothetical protein MAV_0109 [Mycobacterium avium 104], NZ_ABIN01000160_P_8921: predicted protein sequence from *M. intracellulare* genome, NOTNCBI_FOR324_P_3844: predicted protein sequence from *M. fortuitum* genome. Note: protein sequences from *M. marinum* and *M. ulcerans* are shown in these clusters, but peptides were not picked from these species. Amino acid sequence is shown for each protein, with protein sequences identified by NCBI accession number. Numbers under the clusters indicate the amino acid number in the sequence; grey bars under the clusters indicate the degree of similarity between the sequences (high level bars  =  high level of similarity, low level bars  =  low level of similarity).
(TIF)
