# Supplementary figures and images for: Identification of Antigens Specific to Non-Tuberculous Mycobacteria: The Mce Family of Proteins as a Target of T Cell Immune Responses
Source: PLoS One. 2011 Oct 25;6(10):e26434. doi: 10.1371/journal.pone.0026434 (PMC3201954; doi:10.1371/journal.pone.0026434)

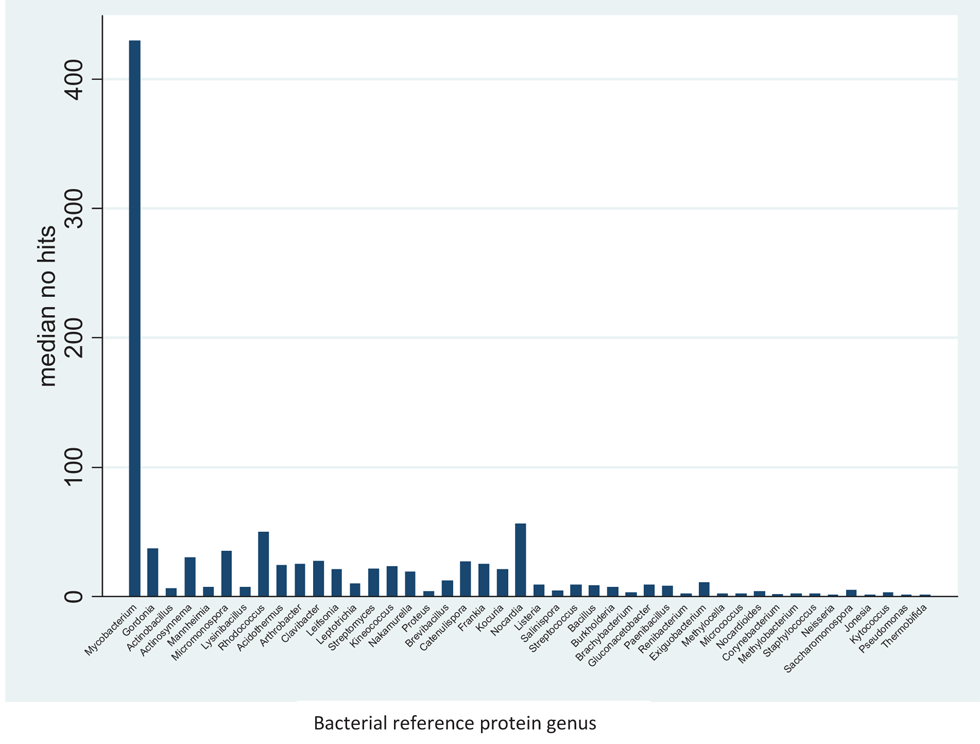

Supplement: Figure S1 — Peptide ‘hits’ on bacterial reference proteins following Protein BLAST. Protein BLAST was carried out on all peptide sequences against all bacterial reference proteins. The resulting hits are shown by bacterial genus. Y axis: median number peptide hits per genus. X axis: bacterial genus. (TIF) [file pone.0026434.s001.tif]

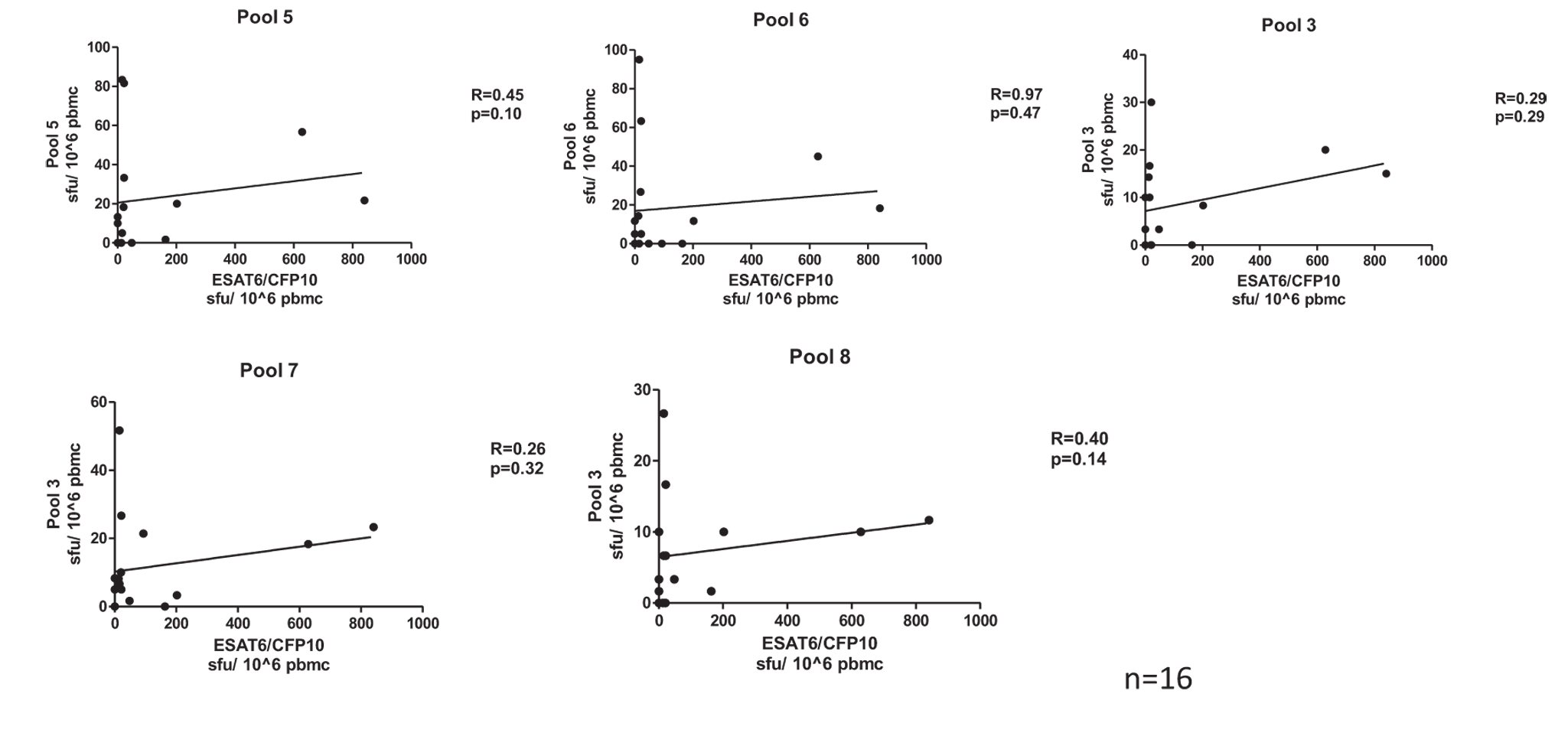

Supplement: Figure S2 — Correlation between response to ESAT-6/CFP-10 peptide pool and NTM-specific peptide pools. Correlation between response to pool of ESAT-6/ CFP-10 peptides and NTM-specific peptide pools, in all healthy South African volunteers. (TIF) [file pone.0026434.s002.tif]

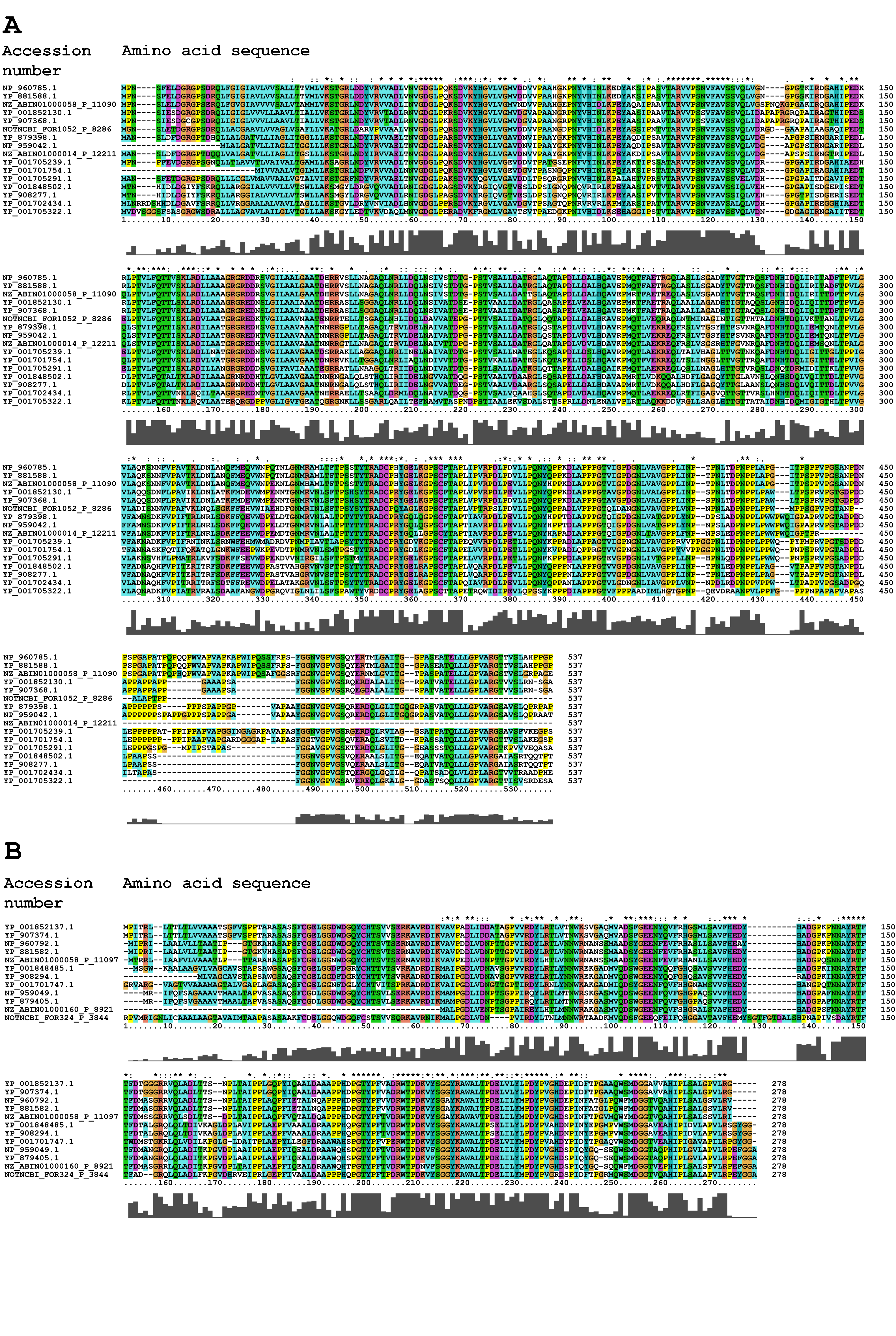

Supplement: Figure S4 — Protein clusters to which immune responses were detected using ex vivo ELISpot and proliferation assays. A. Protein sequences making up cluster A: NP_960785.1: hypothetical protein MAP1851 [Mycobacterium avium subsp. paratuberculosis K-10], YP_881588.1: mce related protein [Mycobacterium avium 104], NZ_ABIN01000058_P_11090: predicted protein sequence from M. intracellulare genome, YP_001852130.1: Mce protein, Mce5A [Mycobacterium marinum M], YP_907368.1: Mce protein, Mce5A [Mycobacterium ulcerans Agy99], NOTNCBI_FOR1052_P_8286: predicted protein sequence from M. fortuitum genome, YP_879398.1: mce related protein [Mycobacterium avium 104], NP_959042.1: hypothetical protein MAP0108 [Mycobacterium avium subsp. paratuberculosis K-10], NZ_ABIN01000014_P_12211: predicted protein sequence from M. intracellulare genome, YP_001705239.1: putative Mce family protein [Mycobacterium abscessus ATCC 19977], YP_001701754.1: putative MCE family protein [Mycobacterium abscessus ATCC 19977], YP_001705291.1: putative Mce family protein [Mycobacterium abscessus ATCC 19977], YP_001848502.1: MCE-family protein Mce6A [Mycobacterium marinum M], YP_908277.1: MCE-family protein Mce6A [Mycobacterium ulcerans Agy99], YP_001702434.1: putative Mce family protein [Mycobacterium abscessus ATCC 19977], YP_001705322.1: putative Mce family protein [Mycobacterium abscessus ATCC 19977]. B. Protein sequences making up cluster B: YP_001852137.1: hypothetical protein MMAR_3871 [Mycobacterium marinum M], YP_907374.1: hypothetical protein MUL_3803 [Mycobacterium ulcerans Agy99], NP_960792.1: hypothetical protein MAP1858 [Mycobacterium avium subsp. paratuberculosis K-10], YP_881582.1: hypothetical protein MAV_2381 [Mycobacterium avium 104], NZ_ABIN01000058_P_11097: predicted protein sequence from M. intracellulare genome, YP_001848485.1: hypothetical protein MMAR_0160 [Mycobacterium marinum M], YP_908294.1: hypothetical protein MUL_4936 [Mycobacterium ulcerans Agy99], YP_001701747.1: hypothetical protein MAB_ [file pone.0026434.s004.tif]
