# Supplementary material for: Identification of Antigens Specific to Non-Tuberculous Mycobacteria: The Mce Family of Proteins as a Target of T Cell Immune Responses
Source: PLoS One. 2011 Oct 25;6(10):e26434. doi: 10.1371/journal.pone.0026434 (PMC3201954; doi:10.1371/journal.pone.0026434)
Supplement: Figure S3 — Peptide pools 5 and 6: constituent proteins and peptides. Showing the identities of proteins making up the clusters in pools 5 and 6, and the amino acid sequences for the peptides derived from them. (PDF) [file pone.0026434.s003.pdf]

Pool 5

Protein accession numbers

NP_960785.1: hypothetical protein MAP1851 [Mycobacterium avium subsp. paratuberculosis K-10]

YP_881588.1: mce related protein [Mycobacterium avium 104]

NZ_ABIN01000058_P_11090: predicted protein sequence from *M. intracellulare* genome

YP_907368.1: Mce protein, Mce5A [Mycobacterium ulcerans Agy99]

NOTNCBI_FOR1052_P_8286: predicted protein sequence from *M. fortuitum* genome

YP_879398.1: mce related protein [Mycobacterium avium 104]

NP_959042.1: hypothetical protein MAP0108 [Mycobacterium avium subsp. paratuberculosis K-10]

NZ_ABIN01000014_P_12211: predicted protein sequence from *M. intracellulare* genome

YP_001705239.1: putative Mce family protein [Mycobacterium abscessus ATCC 19977]

YP_001701754.1: putative MCE family protein [Mycobacterium abscessus ATCC 19977]

YP_001705291.1: putative Mce family protein [Mycobacterium abscessus ATCC 19977]

YP_001702434.1: putative Mce family protein [Mycobacterium abscessus ATCC 19977]

YP_001705322.1: putative Mce family protein [Mycobacterium abscessus ATCC 19977]

Peptide sequences

VKVDAQLMNVGDGLPERADV
GDGLPERADVKFRGMLVGAV
KFRGMLVGAVTSVTPAEDGK
TSVTPAEDGKPNIVHIDLKS
PNIVHIDLKSEHAGGIPSTV
EHAGGIPSTVTARVVPSNVF
TARVVPSNVFAVSSVQLVDN
GDGAGIRNGAIITEDT
RNGAIITEDTKLPTVLFQTT
KLPTVLFQTTTNKLRQVLAA
TERQRGDPPVGLIGVFGEAT
GARLQAILTEFNAMVTASPN
FNAMVTASPNDPSTIAALEK
DPSTIAALEKVSDALSTTSP
VSDALSTTSPRLLDNLENAL
VPLRTLAQKKDDVRGLLSAG
DDVRGLLSAGLHTTGTTATA
IDNHIDQMIGIGTHLTPVVG
VLAQNADKFVPIATRVRALS
PIATRVRALSDAAFANGWDP
DAAFANGWDPGRQVIGLNLI
GRQVIGLNLILSFSPAWTYV
LSFSPAWTYVRDDCPRYGEL
AGPSCTTAPETRQWIDIPEV
TRQWIDIPEVLQPGSYKPPP
DIAPPPGTVFPPPAADIMLH
PPPAADIMLHGTGPNPQE
GTGPNPQEVDRAANPVLP
GPVGSAVEREQLGKALGG
MANSLDFDGRGPTDHQ
LKSTGRLNDYIRVVAELTNV
IRVVAELTNVGDGLPARSDV
GDGLPARSDVKYHGLLVGAV
KYHGLLVGAVDNVIPAAYGK
DNVIPAAYGKPNYVHINLKP
PNYVHINLKPEYAQDIPSAV
EYAQDIPSAVTARVVPSNVF
TARVVPSNVFAVSSVQLVDG
AVSSVQLVDGAPGPSI
APGPSIRNGARIPEDL
RNGARIPEDLQLSTVIFQTT
NNRRGPLLTAGAQLTRVLDE
GAQLTRVLDELNAIVATDPG
PSTVSALLDATRGLQSTAP
ATRGLQSTAPDLVDVLHDAV
DLVDVLHDAVRPMQTLVEKR
RPMQTLVEKREQFRSLVTGS
EQFRSLVTGSYHTFSVNRQA
YHTFSVNRQAFDNHTDQLIE
FDNHTDQLIEMTQNLTPVLG
MTQNLTPVLGVFAMNSDKFV
VFAMNSDKFVPIFTRLNRLS
PIFTRLNRLSDKFFQEVWDP
DKFFQEVWDPELDTGNMRVN
ELDTGNMRVNLALTPTYTYT
LALTPTYTYTRADCPRYGQL
QGPSCFTAPTIAVRPDLPEV
IAVRPDLPEVLLPQNYHPPT
DLAPPPGTQIGPDGNLVATG
PPLYNPDPSLADPNPPLP
SLADPNPPLPWWPWQIGPAP
WWPWQIGPAPRVPGTADPDD
VAPAAYGGNVGPVGSQRERD
QLGLITGQGRPASVATQLLL
AVSSVQLVGNGPGSKI
GPGSKIRDGAHIPEDK
PASEATQLLLGPVARGTTVS

Pool 6

Protein accession numbers

NP_960792.1: hypothetical protein MAP1858 [Mycobacterium avium subsp. paratuberculosis K-10]

YP_881582.1: hypothetical protein MAV_2381 [Mycobacterium avium 104]

NZ_ABIN01000058_P_11097: predicted protein sequence from *M. intracellulare* genome

YP_001701747.1: hypothetical protein MAB_1003c [Mycobacterium abscessus ATCC 19977]

NP_959049.1: hypothetical protein MAP0115 [Mycobacterium avium subsp. paratuberculosis K-10]

YP_879405.1: hypothetical protein MAV_0109 [Mycobacterium avium 104]

NZ_ABIN01000160_P_8921: predicted protein sequence from *M. intracellulare* genome

NOTNCBI_FOR324_P_3844: predicted protein sequence from *M. fortuitum* genome

PVALAPLASADPPPPPPDP
AAPAVDAGPPPDNGLVASA
PPDNGLVASAEPGVVKTPDG
EPGVVKTPDGWTLTVSAKDE
WTLTVSAKDESQLPVAPLTT
SQLPVAPLTTAASSREYLVG
AASSREYLVGATFTGTVTGS
GSTTLNGGTLDTGYQIGCGI
DTGYQIGCGIELGQVRL
STLAGIIPTGVS
LAGIIPTGVSMPISGTIEIH
MPISGTIEIHAKPGTVTNVS
AKPGTVTNVSVNKKSFKAAP
VNKKSFKAAPVRVTVKDVHI
VRVTVKDVHIKIDGCVGQSF
LRSYATLTSSTTDTDDVVAY
TTDTDDVVAYYGVTKAV
MTVAGSNETLLPVAPLTT
TLLPVAPLTTAISSREYLVG
GTFTGKVSGGGKTKLTGGTL
GKTKLTGGTLEAGAQIGCGI
EAGAQIGCGIISDETEI
ISDETEINPGLSFTPGI
NPGLSFTPGIKIPFTGS
KIPFTGSAGDASLG
LKPGTVTIVPIDKKSFKGTG
IDKKSFKGTGTRVTITGVRI
TRVTITGVRIKFDQCAGQSF
KFDQCAGQSFIRTYATLTSS
IRTYATLTSSTDNTDDVITY
TDNTDDVITYLGVTKAV
FGLLAAVSVACAASATPAWA
EPAADLVESTPPAHLTTPDG
PPAHLTTPDGWHLTVSASDE
TRAAVPPLTTAISSREYVVG
AISSREYVVGGTYRGSMTGP
GTYRGSMTGPGGGDDLRGTL
GGGDDLRGTLEAGYQIGCGI
EAGYQIGCGIDMSTSNGVAL
DMSTSNGVALTGTAGLNGSI
LKPGIINLIPVTEKEFKGTE
VTEKEFKGTEPWVMISNFRV
PWVMISNFRVKIDGCVGQSF
KIDGCVGQSFIRSYAVLTRS
TDESEAVLAWYGTTKVV
RRSRTMLKTLAALTAAYSLA
AATPLAGANPTEGQPVPVMP
WVLSVAASDETQQSVAPLTT
TQQSVAPLTTALSSREYVVG
ALSSREYVVGGSFMGTVKGA
EAGYQIGCGVNADVVDA
NADVVDARFGGSLTPSG
RFGGSLTPSGSL
GGSPSLGGNLFAQIRPT
GNLFAQIRPTLKPGTTTIVP
LKPGTTTIVPVTKMDYKGDS
VTKMDYKGDSARVTITALRI
ARVTITALRIKIDSCVGQSF
KIDSCVGQSFIRSYATFTSA
IRSYATFTSATADTQDVVSY
TADTQDVVSYVGVTKAV
MASGEPTVVTTPDD
EPTVVTTPDDWTLKLAATGE
WTLKLAATGETQLPIPPLTT
TQLPIPPLTTALSSREYLAG
ALSSREYLAGGTFTGSVEGA
GRTTLKGGTLEAGYQIGCGI
EAGYQIGCGIELSAVRL
VPIQGNVEVRPKPGEVLNVS
PKPGEVLNVSVTQKKFEGTK
VTQKKFEGTKSRVTLKDVHI
SRVTLKDVHIKIDGCVGQSF
LRSYAKLTSATTGTQDIVAY
TTGTQDIVAYYGVTKTV
MAAVCLALSLPTAPTTGA
MAIAVCLAMSVIAAPSADS
SVIAAPSADSEPQLGPA
EPQLGPAPPDAA
PPAGAAVPSNPPAILNTPDG
PPAILNTPDGWTLGLGAKDE
WTLGLGAKDEMQVPVAPLTT
MQVPVAPLTTAISSREYMSS
